# Genomic Evidence Suggests Viral Persistence of SARS-CoV-2 for 386 Days in Health Worker: A Case Report from Santiago of Chile

**DOI:** 10.3390/idr14060096

**Published:** 2022-11-30

**Authors:** Claudio Acuña-Castillo, Kevin Maisey, Mabel Vidal, Carlos Barrera-Avalos, Ailen Inostroza-Molina, Roberto Luraschi, Eva Vallejos-Vidal, Daniel Valdés, Mónica Imarai, Felipe E. Reyes-López, Ana María Sandino

**Affiliations:** 1 Centro de Biotecnología Acuícola, Facultad de Química y Biología, Universidad de Santiago de Chile, Santiago 9160000, Chile; 2 Departamento de Biolgía, Facultad de Química y Biología, Universidad de Santiago de Chile, Santiago 9160000, Chile; 3 Department of Computer Science, University of Concepcion, Concepción 4070409, Chile; 4 Centro de Nanociencia y Nanotecnología CEDENNA, Universidad de Santiago de Chile, Santiago 9160000, Chile; 5 Núcleo de Investigación Aplicada en Ciencias Veterinarias y Agronómicas, Facultad de Medicina Veterinaria y Agronomía, Universidad de Las Américas, Santiago 7500975, Chile

**Keywords:** COVID-19, SARS-CoV-2, persistence, pandemic, genomic surveillance

## Abstract

The COVID-19 pandemic continues to affect several countries. One of the best ways to control its spread is the timely identification of infected patients for isolation and quarantine. While an episode of infection lasts an average of 8–10 days from the onset of symptoms, there is literature describing long-lasting viral persistence events. Here, we report a case of persistence of SARS-CoV-2 for 386 days in a health worker from Santiago de Chile. Our study could be one of the longest reported viral persistence events. RNA sequencing analyses indicated that the first positive diagnosis (8 June 2020) corresponded to a SARS-CoV-2 variant belonging to Clade Nextstrain 20A. Three hundred eighty-six days later (23 September 2021), the second positive result reached the same viral variant (Clade 20A) but without presence or circulation in Chile since May 2021. Both sequencing coverages showed an identity of 99.21%, with some mutations related to the severity of the disease (ORF1b:P314L) and more infectivity (S:D614G). This work reinforces the idea of implementing an RT-qPCR or rapid antigen test once the quarantine is fulfilled to ensure viral absence, identify potential persistence, and, consequently, minimize the risk of local outbreaks of SARS-CoV-2 infection.

## 1. Introduction

The pandemic of the coronavirus disease 2019 (COVID-19) has caused around 6.2 million deaths worldwide and infected 500 million [1]. Vaccines have effectively controlled infections and fatalities associated with the disease. However, the appearance of new variants and lack of global coverage can generate new infections. Therefore, one of the best ways to control its spread is the timely identification of infected patients for isolation and quarantine. A typical episode of illness lasts an average of 8–10 days from the onset of symptoms and 20 days in immunocompromised patients [2]. However, long-lasting viral persistence events have been reported. For example, Truong et al., 2021, indicate a viral persistence of 165 days in a male patient with cancer therapy [3], while Ma et al., 2022, described six cases of viral persistence of up to 106 days in young asymptomatic patients [4]. Although some reports indicate that it is unlikely that after ten days the virus will continue to infect [5], it has been reported that the virus can remain infective even for over three months [6] after the manifestation of symptoms and the quarantine period. Under this long-lasting infection scenario, the persistence of SARS-CoV-2 increases the probability of generating contagion outbreaks even in clinically recovered patients, being an underestimated event that may provoke community outbreaks. This study suggests a viral persistence of 386 days, one of the longest reported. SARS-CoV-2 genome-wide analysis indicated that the SARS-CoV-2 variant in the second COVID-19 diagnosis was classified in the clade Nextstrain 20A, with 0% circulation in Chile, from May 2021 [7]. Interestingly, both samples showed a 99.21% identity with each other. Our study suggests particular attention to these ongoing events, as it may be vital in designing genomic surveillance protocols and public health policies for this and other future pandemics.

## 2. Materials and Methods

Case description. The patient was a 29 years old female health worker (nurse technician) with no comorbidities and reported no condition of immunosuppression. The RNA extraction and RT-qPCR COVID-19 diagnosis were produced as we described previously [8]. The SARS-CoV-2 detection of all nasopharyngeal swab samples (NPSs) was carried out by detecting the ORF1ab gene using an RT-qPCR kit (TaqMan™ 2019 nCoV Assay Kit v1, Thermo Fisher Scientific, Cat. No. A47532) and the one-step strategy, in the same way as previously described [9]. She was diagnosed as COVID-19 positive by RT-qPCR (NPSs) during routine control on 8 June 2020 (0806-191), with no symptomatology. Five RT-qPCR tests were performed after her first diagnosis on 22 June 2020, 23 October 2020, 14 April 2021, 9 July 2021, and 23 September 2021 (230921-099). The last sample RT-qPCR was positive for COVID-19 and related to symptomatology, which included rhinorrhea, sore throat, fever, diarrhea, asthenia, and mild headache. After the second positive COVID-19 diagnosis on 23 September 2021 and completing the patient’s quarantine for eleven days, followed by symptom relief, the patient underwent two additional RT-qPCR tests. The first was on 25 January 2022 (116 days later, No Cq), while the second was on 6 May 2022 (225 days later, Cq 37.52), which showed negative results. The patient, during the analysis, was vaccinated. The first dose was on 17 June 2021 (374 days after the start and 98 days before the end of the study on 23 September 2021). The second dose was on 15 July 2021 (70 days before the end of the study). At the same time, she had two booster doses, which were on 22 November 2021 and 17 May 2022, both dates outside the study period. The vaccination schedule for the first two doses was performed with the CoronaVac vaccine from Sinovac Life Sciences. The booster doses (3rd and 4th doses) were Pfizer-BioNTech vaccines.

Sequencing and analysis. The 0806-191 and 230921-099 positive samples were provided by the Laboratory of Virology, Centro de Biotecnología Acuicola, Universidad de Santiago de Chile, sequenced and analyzed by the Center for Genome Regulation (CGR). First, the cDNA synthesis of the samples was performed using the High-Capacity RNA-to-cDNA kit (Applied Biosystems) and the amplification of the samples was performed using the ARTIC v3 protocol with modifications. Briefly, samples were amplified using the 98 primers set with the GoTaq Green Master Mix enzyme (Promega), and the genomic library was constructed using the Ligation Sequencing Kit SQK-LSK109 (Oxford Nanopore) following the manufacturer’s instructions, but processed and sequenced independently (without barcodes) to increase and know the exact number of readings per sample. Each sample was sequenced to reach 1 Gigabase (Gb) (approximately 3 h of sequencing) without the base-calling process. The Nanopore sequencing was transformed from raw fast5 files to a recognizable base-called format (fastq) using the ONT Guppy base caller [Guppy] https://nanoporetech.com (accessed on 11 July 2022) (CPU mode). After base-calling, all reads were quality-checked and assembled using a custom version of the recommended ARTIC MinION pipeline [ARTICMINION] https://artic.network/ncov-2019 (accessed on 15 July 2022). The consensus assembly genomes were aligned to the reference SARS-CoV-2 genome using Nucmer [MUMmer] and the SNVs were predicted using Nucmer tool show-snps. Then, the SNVs were annotated using SnpEff [SNPEFF] within the SARS-CoV-2 database. All computations were run at the National Laboratory for High-Performance Computing (www.nlhpc.cl, accessed on 15 July 2022). In addition, a clade assignment was performed using NextClade version 1.9.0 software to compare and visualize our samples globally. Library preparation and sequencing of the positive samples are detailed on the Bioproject (NCBI) database under the accession number PRJNA817604 (BioSample accession numbers SAMN26801552 and SAMN26801553 for samples 080620-191 and 230921-099, respectively). The quality of the sequencing was determined with the MultiQC software. The analysis of the sequencing coverage according to the SARS-CoV-2 genome was carried out by FastQC software for both samples.

Phylogenetic tree generation. The evolutionary history was inferred by using the Maximum Likelihood method and JTT matrix-based model [10]. The tree with the highest log likelihood (−397,625.64) is shown. The percentage of trees in which the associated taxa clustered together is shown next to the branches. The initial tree(s) for the heuristic search were obtained automatically by applying Neighbor-Join and BioNJ algorithms to a matrix of pairwise distances estimated using the JTT model and then selecting the topology with superior log likelihood value. The tree is drawn to scale, with branch lengths measured in the number of substitutions per site (next to the branches). This analysis involved seven amino acid sequences. There was a total of 29,903 positions in the final dataset. The evolutionary analyses were conducted in MEGA X [11,12].

Viral load. To estimate the viral load of the samples, we constructed a standard curve by creating serial 1/10 dilutions using the positive control TaqMan 2019-nCoV Control Kit v1 (104 copies/µL) (Thermo Fisher Scientific, Cat. No. A47533). An antilogarithm of the following equation of the line (y = −3.07X + 40.2)/2 was used to calculate the viral load. The Cq captured in the NPSs analysis was replaced in “X”.

Ethics statement. This study was authorized by the Ethical Committee of the University of Santiago of Chile (No. 226/2021) and the Scientific Ethical Committee of the Central Metropolitan Health Service, Ministry of Health, Government of Chile (No. 370/2021) and it follows the Chilean law in force.

## 3. Results

**Figure 1:**
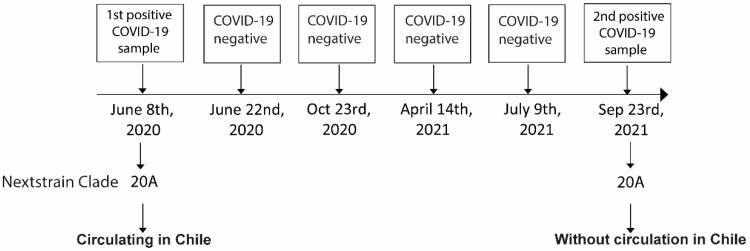
Timeline of RT-qPCR tests and genome analysis of SARS-CoV-2. Timeline of the RT-qPCR tests performed on the health worker. The Nextstrain classification lineage of the positive samples (0806-191 and 230921-099) are shown. According to official reports from the Chilean Ministry of Health, Clade 20A has not been circulating in Chile since May 2021.

**Figure 2:**
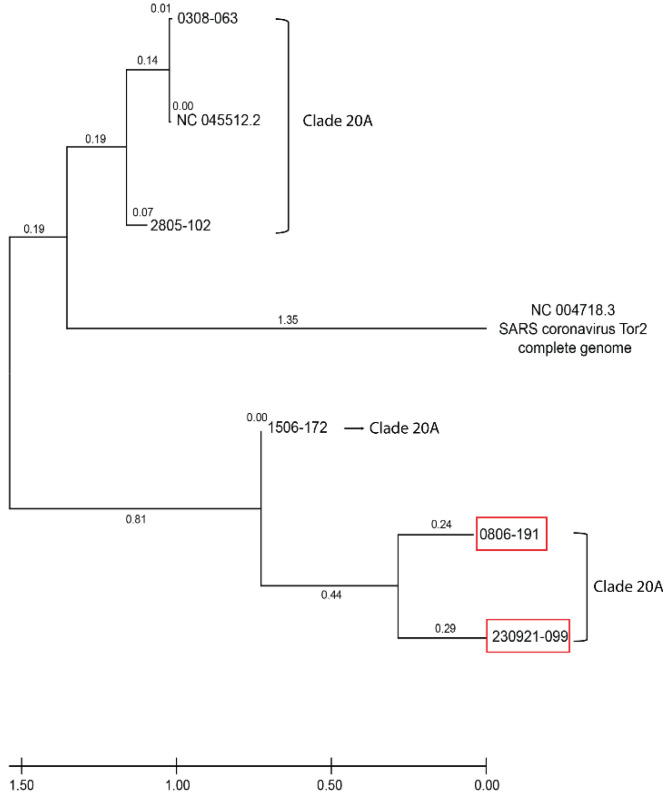
Phylogenetic analysis of samples 0806-191 and 230921-099. Three NPSs were added: 1506-172, 2805-102, and 0308-063, all belonging to the same Clade 20A. The new samples belong to the samples sequenced and stored in our Laboratory of Virology from the Centro de Biotecnología Acuícola, Universidad de Santiago de Chile. The samples analyzed in this study are highlighted with a red box.

A 29-year-old female healthcare worker without comorbidities was diagnosed as COVID-19 positive by RT-qPCR in a nasopharyngeal swap sample (NPSs) during routine control on 8 June 2020 (0806-191) (Cq = 31.85; 2.62 × 102 copies/µL). After 14 days, on 22 June 2020, the patient turned RT-qPCR negative (No Cq). After 137 days, on 23 October 2020, the patient was diagnosed negative with no viral gene amplification (No Cq). The patient was again diagnosed negative 176 days later (14 April 2021), but with a very slight amplification (Cq = 36.33) and determined under the Limit value of Detection (LoD). Then, 83 days later, on 9 July 2021, the patient was diagnosed as negative again (No Cq). Finally, 14 days later, the patient was diagnosed positive for COVID-19 on 23 September 2021 (230921-099), with Cq = 34.03 and a viral load of 5.11 × 101 copies/µL. The symptomatology included rhinorrhea, sore throat, fever, diarrhea, asthenia, and mild headache. The analysis using RNA sequencing of both positive samples (0806-191 and 230921-099) indicated that both sequences belong to the same clade Nextstrain 20A lineage (Figure 1). Importantly, in September 2021, this clade was no longer reported in circulation in Chile. The sequencing of samples 0806-191 and 230921-099 (99.1% and 37.4% coverage of the entire viral genome, with 99.9% and 99.7% identity upon the reference SARS-CoV-2 genome, respectively) showed a 99.21% identity between them. Figure 2 shows a phylogenetic tree with the samples analyzed in this study and three additional samples from clade 20A. We observe that samples 0806-191 and 230921-099 (highlighted in the red box) are part of the same cluster and present a minimal divergence between them. The closest sample is 1506-172, a sample from June 2020, while sample 0308-063 is the furthest, even belonging to the same Clade 20A, from August 2020. The quality of both sequencings is shown in Supplementary Figure S1. The NGS coverage across the SARS-CoV-2 genome of samples 0806-191 and 230921-099 are shown in Supplementary Figures S2 and S3.

The genomic sequence analysis for samples 0806-191 identified seven single nucleotide variants (SNVs) and 230921-099 showed fifteen SNVs compared with the reference genome. On the other hand, three deletions were noted for the sample 230921-099 relative to the reference genome. Notably, four SNVs were shared between both samples. Sample 0806-191 had three SNVs not seen in sample 230921-099, whereas sample 230921-099 had eleven SNVs absent in sample 0806-191 (Table 1). Some of these genomic mutations generate amino acid changes, which are shown in Table 1.

## 4. Discussion

A series of previous works have documented the long-term viral persistence of SARS-CoV-2. Zahn et al., 2021, reported 37 days in patients with mild symptoms. These were clinically discharged from ten days after the onset of symptoms; however, they were still infected with the virus [13]. Other reports have described viral persistence for 42 days with an infective capacity in an asymptomatic patient [14]. In addition, the persistence of SARS-CoV-2 can also be related only to the presence of viral RNA. For example, SARS-CoV-2 RNA shedding has been detected 63, 83, and 106 days after the onset of symptoms in the upper respiratory tract [4,15,16]. Although the persistence of RNA and infectious viruses is generally related to immunosuppressed patients, older adults, or those who developed severe COVID-19 with a high viral load also have a longer persistence time [17,18]. The average infection and viral clearance time do not last more than ten days [2]. However, no report has suggested viral RNA persistence of 386 days.

In this study, we sequenced the SARS-CoV-2 genome from the NPSs of a health worker on 8 June 2020, the first year of the pandemic in Chile. The results and analysis indicated that it belonged to the Clade Nextstrain 20A lineage, one of the reported SARS-CoV-2 circulating in Chile. After a series of RT-qPCR tests with negative results under the LoD, the patient tested positive 386 days later (23 September 2021). Genome sequencing analyses indicated the presence of the same initial variant, which interestingly had 0% circulation in Chile in September 2021, according to the epidemiological report of the Ministry of Health of Chile [19], with an identity of 99% between the first and last sequenced sample. Precisely on that date, 55–60% of the circulating variants were Delta (21A), about 20% were Mu (21H), and 20% were Gamma (20J) [19]. Furthermore, all mutations in the second sample could suggest a possible viral genomic change within the same patient at 386 days, with a silent persistence scenario or a long latency or inactivity [20]. In our study, if there was a viral latency period, it was related to viral reactivation. Patients without immunosuppression, who completed their vaccination schedule or were recently infected, have at least 3–5 months of protection before a reinfection period [21,22]. As in our case, the patient completed her vaccination schedule 70 days before the second episode of a positive diagnosis. Previous reports have documented reactivation as a form of viral persistence. For example, one study reported two cases of patients who completed their quarantine with clinical discharge and two negative RT-qPCR tests. However, two weeks later, both tested positive for COVID-19, with a low probability of viral reinfection due to the short time elapsed [23]. Tang X. et al. (2021) pointed out that positive tests within at least 15 days correspond to viral reactivation [24]. If this were the case for viral reactivation, it would be the most extended period reported to date after a latency period.

After sequencing both positive samples with 386 days of difference, some important mutations were affected, such as ORF1b:P314L and S:D614G. The first mutation was related to a greater spread and severity of the disease in patients in Europe compared to geographic locations where they were not frequent, such as in Asia [25], while the second is related to an increase in the ineffectiveness of the virus on the cells [26]. This antecedent may be indirectly related to immunological evasion by these viral variants, with a long persistence on the host. Despite this evidence, the reinfection scenario is also possible, considering that there were no consecutive positive results and the broad spectrum of days from one test to another, where natural immunity decreases, causing more new infections [27], independent of no register of that variant in Chile. However, the interesting point is that the patient completed the CoronaVac vaccination schedule 70 days before the second positive COVID-19 diagnosis. Previous studies have documented that complete vaccination of two doses of CoronoVac protects patients for between 3 and 5 months against SARS-CoV-2 infection [22]. This is similar to that indicated by studies for other vaccines that suggest they produce around six months of protection even in elderly patients [28,29].

One limitation is the lack of an assay to recover the virus from the analyzed samples and test its infectivity in vitro as previously performed [30]. In addition, the fact that the patient studied is a health worker increases the likelihood of reinfection rather than persistence, even with a virus that has not been reported in Chile for months. Interestingly, the negative results in the middle of the period corresponding to Cq values above the detection limit of the technique, which cannot rule out that the virus is present, especially at a viral Cq of 36.33 (14 April 2021). Another limitation is important to highlight. Although the coverage percentage of the second positive sample meets a high-quality score in sequencing, it has a coverage of 37.4%, which is low compared to the coverage of the first sequence studied (close to 99%). Although both sequencing analyses indicate belonging to the same Clade 20A, it could negatively affect the inferred similarity between the first and second samples collected.

On the other hand, persistent SARS-CoV-2 RNA shedding in the respiratory tract is possible. However, we would not have detected fourteen mutations between the first and second samples in this case. Although some tests are missing to ensure viral persistence, such as the verification of infectivity in primary cell cultures or positive RT-qPCR tests as reported in other studies, we could be facing the highest persistence of SARS-CoV-2 reported to date. In addition, the long continuance of infective RNA or SARS-CoV-2 could explain the long-lasting symptoms related to COVID-19 [31]. Our study, together with others reported in the literature, suggests maintaining active genomic surveillance and paying particular attention to cases of viral persistence and their potential incidence in the generation of small outbreaks of local contagion or its eventual capacity to act as a viral reservoir.

## Figures and Tables

**Table 1:** SNVs of the first (0806-191) and second (230921-099) positive samples for COVID-19 compared to the reference SARS-CoV-2 genome. The amino acid changes are related to the corresponding genetic mutation and SARS-CoV-2 gene.

0806-191			230921-099		
Genome Location	SNVs	Amino Acid Substitution	Genome Location	SNVs	Amino Acid Substitution
241	C->T	n/a	241	C->T	n/a
3037	C->T	n/a			
			6402	C->T	n/a
6479	G->A	n/a	6479	G->A	n/a
6706	C->T	ORF1a:V2072I			
			8043	C->A	n/a
			10511	G->C	n/a
14408	C->T	ORF1b:P314L	14408	C->T	ORF1b:P314L
			15451	G->T	n/a
			17699	T->C	ORF1b:I1411T
			18457	C->T	ORF1b:P1664S
			22088	C->T	S:L176F
23403	A->G	S:D614G			
27137	A->G	n/a	27137	A->G	n/a
			27604	G->A	ORF7a:V71I
			27638	T->C	ORF7a:V82A
			27670	G->T	ORF7a:V93F
			27752	C->T	ORF7a:T120I
			Δ 28248-28253	GATTTC	n/a
			Δ 28273	A	n/a
			Δ3590	A	n/a

n/a = not applicable. The similarities (gray) and differences (white) of mutations between both analyzed samples are shown.

## Data Availability

The data that support the findings of this study are available in the NCBI database BioSample (https://www.ncbi.nlm.nih.gov/biosample/, accessed on 15 August 2022); SAMN26801552 and SAMN26801553 for samples 080620-191 and 230921-099, respectively).

## Supplementary Materials

The following supporting information can be downloaded at: https://www.mdpi.com/article/10.3390/idr14060096/s1, Figure S1: Sequence quality score of the two positive samples sequenced (0806-191 and 230921-099). Mean sequence quality (Phred Score) y-axis. The graph is divided into the sequencing of excellent quality (green), acceptable quality (orange), and poor quality (red) concerning the number of bp on the x-axis. Both samples show excellent sequencing quality, Figure S2: Coverage of NGS in the SARS-CoV-2 genome of sample 0806-191. Sequence content for all bases (T in red, C in blue, A in green, and G in black) and their position in the read (bp) of sample 0806-191 (at 99.1% coverage). The coverage of the sequenced genes of the SARS-CoV-2 genome (29 kbp) is shown, Figure S3: Coverage of NGS in the SARS-CoV-2 genome of sample 230921-099. Sequence content for all bases (T in red, C in blue, A in green, and G in black) and their position in the read (bp) of sample 230921-099 (at 37.4% coverage). The coverage of the sequenced genes of the SARS-CoV-2 genome (29 kbp) is shown.
