# Supplementary material for: Genomic Evidence Suggests Viral Persistence of SARS-CoV-2 for 386 Days in Health Worker: A Case Report from Santiago of Chile
Source: Infect Dis Rep. 2022 Nov 30;14(6):971–8. doi: 10.3390/idr14060096 (PMC9778366; doi:10.3390/idr14060096)
Supplement: Supplementary file 1 [file idr-14-00096-s001.zip › idr-1894568-supplementary.pdf]

SUPPLEMENTARY MATERIAL

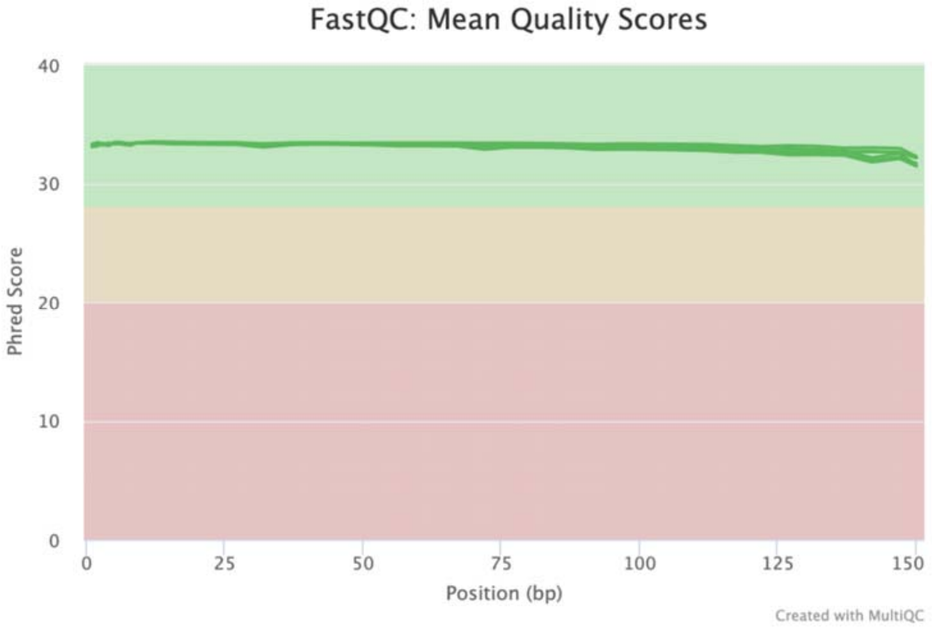

Supplementary Figure S1. Sequence quality score of the two positive samples sequenced (0806-191 and 230921-099). Mean sequence quality (Phred Score) y-axis. The graph is divided into the sequencing of excellent quality (green), acceptable quality (orange), and poor quality (red) concerning the number of bp on the x-axis. Both samples optimum sequencing quality. The quality analyzes were created with the MultiQC software

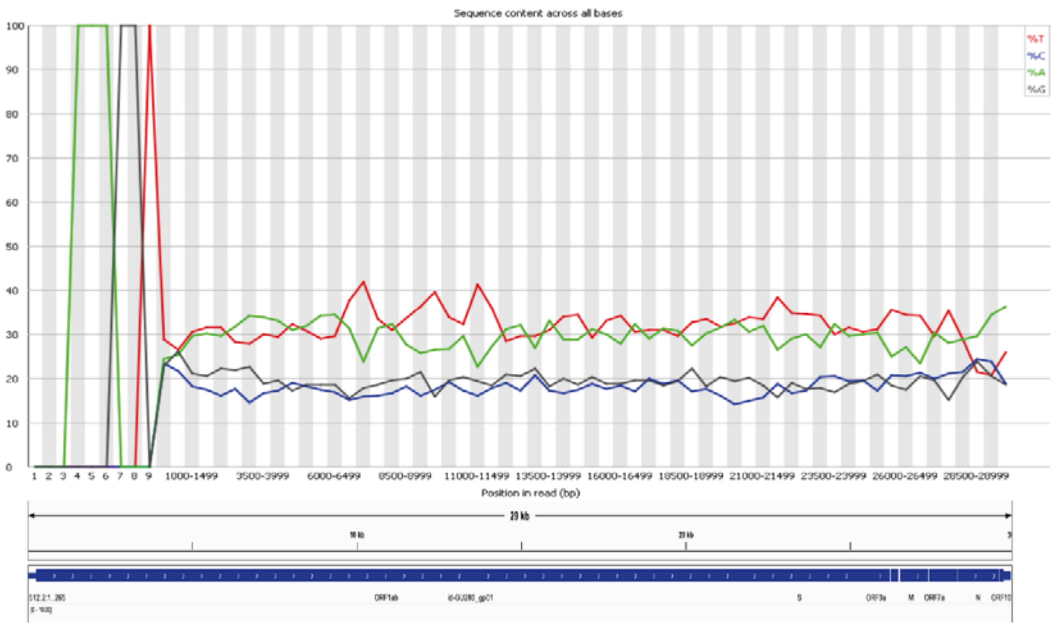

Supplementary Figure S2. Coverage of NGS in the SARS-CoV-2 genome of sample 0806-191. Sequence content for all bases (T in red, C in blue, A in green, and G in black) and their position in the read (bp) of sample 0806-191 (at 99.1% coverage). The coverage of the sequenced genes of the SARS-CoV-2 genome (29 kbp) is shown. The quality analyzes were created with the FastQC software.

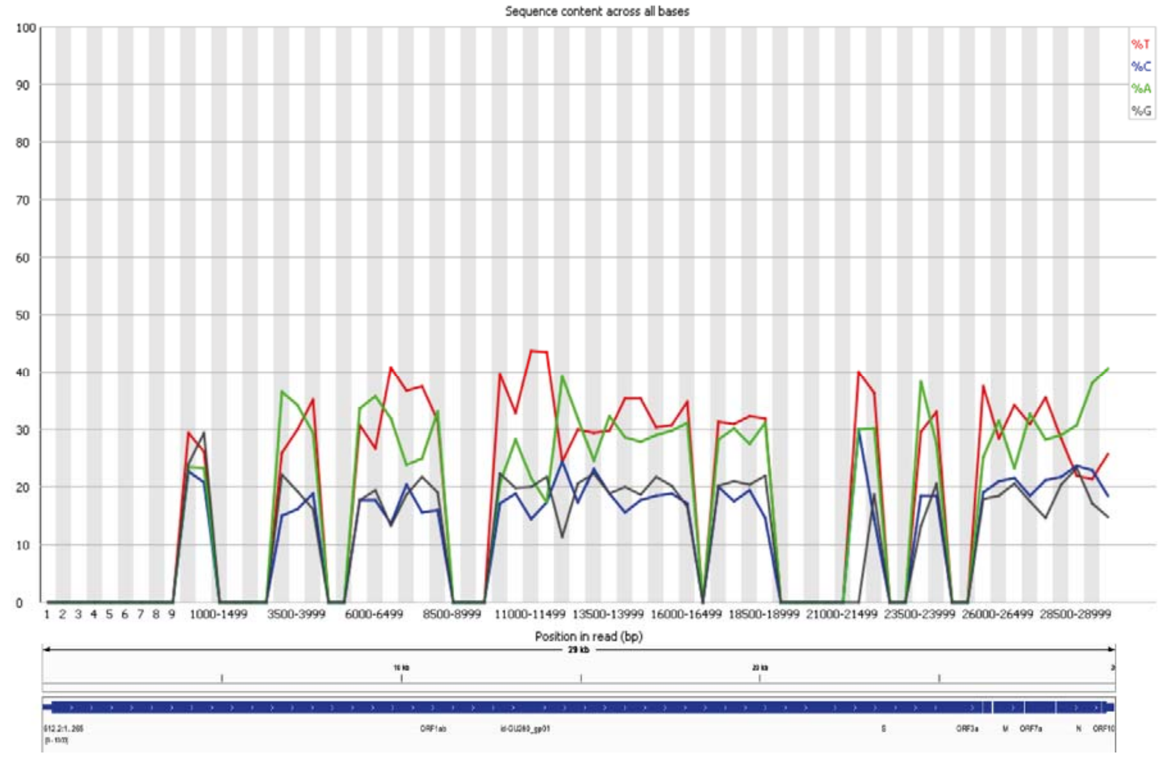

Supplementary Figure S3. Coverage of NGS in the SARS-CoV-2 genome of sample 230921-099. Sequence content for all bases (T in red, C in blue, A in green, and G in black) and their position in the read (bp) of sample 230921-099 (at 37.4% coverage). The coverage of the sequenced genes of the SARS-CoV-2 genome (29 kbp) is shown. The quality analyzes were created with the FastQC software.
